# The incidence of diabetes mellitus and diabetic retinopathy in a population-based cohort study of people age 50 years and over in Nakuru, Kenya

**DOI:** 10.1186/s12902-017-0170-x

**Published:** 2017-03-23

**Authors:** Andrew Bastawrous, Wanjiku Mathenge, Kevin Wing, Madeleine Bastawrous, Hillary Rono, Helen A. Weiss, David Macleod, Allen Foster, Tunde Peto, Peter Blows, Matthew Burton, Hannah Kuper

**Affiliations:** 1 International Centre for Eye Health, Clinical Research Department, London School of Hygiene & Tropical Medicine, London, UK; 2 Rwanda International Institute of Ophthalmology and Dr Agarwal’s Eye Hospital, London, Rwanda; 3 Department of Non-Communicable Disease Epidemiology, London School of Hygiene & Tropical Medicine, London, UK; 4 Nuffield Department of Primary Care, University of Oxford, Oxford, UK; 5 Kitale Eye Unit and Trans Nzoia County, London, Kenya; 6 MRC Tropical Epidemiology Group, Department of Infectious Disease Epidemiology, London School of Hygiene & Tropical Medicine, London, UK; 7 NIHR BMRC at Moorfields Eye Hospital NHS Foundation Trust and UCL Institute of Ophthalmology, London, UK; 8 Centre for Public Health, Queen’s University Belfast, London, UK; 9 Moorfields Eye Hospital NHS Foundation Trust, London, UK

## Abstract

**Background:**

The epidemic rise of diabetes carries major negative public health and economic consequences particularly for low and middle-income countries. The highest predicted percentage growth in diabetes is in the sub-Saharan Africa (SSA) region where to date there has been no data on the incidence of diabetic retinopathy from population-based cohort studies and minimal data on incident diabetes. The primary aims of this study were to estimate the cumulative six-year incidence of Diabetes Mellitus (DM) and DR (Diabetic Retinopathy), respectively, among people aged ≥50 years in Kenya.

**Methods:**

Random cluster sampling with probability proportionate to size were used to select a representative cross-sectional sample of adults aged ≥50 years in 2007-8 in Nakuru District, Kenya. A six-year follow-up was undertaken in 2013–14. On both occasions a comprehensive ophthalmic examination was performed including LogMAR visual acuity, digital retinal photography and independent grading of images. Data were collected on general health and risk factors. The primary outcomes were the incidence of diabetes mellitus and the incidence of diabetic retinopathy, which were calculated by dividing the number of events identified at 6-year follow-up by the number of people at risk at the beginning of follow-up. Age-adjusted risk ratios of the outcomes (DM and DR respectively) were estimated for each covariate using a Poisson regression model with robust error variance to allow for the clustered design and including inverse-probability weighting.

**Results:**

At baseline, 4414 participants aged ≥50 years underwent complete examination. Of the 4104 non-diabetic participants, 2059 were followed-up at six-years (50 · 2%). The cumulative incidence of DM was estimated at 61 · 0 per 1000 (95% CI: 50 · 3–73 · 7) in people aged ≥50 years. The cumulative incidence of DR in the sample population was estimated at 15 · 8 per 1000 (95% CI: 9 · 5–26 · 3) among those without DM at baseline, and 224 · 7 per 1000 (116.9–388.2) among participants with known DM at baseline. A multivariable risk factor analysis demonstrated increasing age and higher body mass index to be associated with incident DM. DR incidence was strongly associated with increasing age, and with higher BMI, urban dwelling and higher socioeconomic status.

**Conclusions:**

Diabetes Mellitus is a growing public health concern with a major complication of diabetic retinopathy. In a population of 1 · 6 million, of whom 150,000 are ≥50 years, we estimated that 1650 people aged ≥50 develop DM per year, and 450 develop DR. Strengthening of health systems is necessary to reduce incident diabetes and its complications in this and similar settings.

## Background

The number of adults with Diabetes Mellitus (DM) in Africa is predicted to double from 12 · 1 million in 2010 to 23 · 9 million in 2030 based on projections from prevalence data [1]. The epidemic rise of DM carries major public health and economic consequences for the continent, particularly given it is home to some of the fastest growing economies and most rapid transitions in lifestyles conducive to DM ([http://unstats.un.org/unsd/methods/m49/m49regin.htm - least]). Currently, there are few incidence data from low and middle-income settings, particularly sub-Saharan Africa (SSA), making it difficult to plan screening and treatment services [2, 3].

DM causes visual impairment through cataract and diabetic retinopathy (DR) [4], a progressive disease of the retinal microvasculature. DR is not yet a leading cause of blindness in sub-Saharan Africa, responsible for just 2.8% of blindness [5]. However, it is likely to become an increasingly important cause of blindness and visual impairment, with the increasing prevalence of DM in SSA, and improving control of other leading causes of visual impairment such as cataract, uncorrected refractive error and trachoma. Population-based incidence data for DR are lacking for SSA, although some clinical follow-up data are available [6].

Current strategies to control DM in SSA focus on health system strengthening to enable a public health approach to both prevent the onset of DM and create awareness of the consequences of DM, including sight loss [7]. In addition, efforts are also being scaled-up for the identification of people with DM , and their enrolment in treatment programmes [8]. Systematic DR screening in SSA is currently very limited, with only a small number of locations having an active programme [9].

Data on the incidence and progression of DM and DR are needed to estimate the current and future burden of these conditions in order to inform service development, and future research. The primary aims of this study were to estimate the cumulative six-year incidence of DM and DR in people aged ≥50 years in Kenya. A secondary aim was to identify risk factors for each of these outcomes.

## Methods

The fieldwork was carried out in Nakuru district, Kenya, which has a population of 1.6 million [accurate as of 2009], one third of which is urban. Nakuru is broadly representative of Kenya in terms of ethnic diversity and economic activities. The baseline survey took place from January 2007 to November 2008, and the follow-up survey from November 2012 to March 2014. Full details of the methods at baseline and follow-up are presented elsewhere [10–12].

### Sampling strategy and recruitment at baseline

We selected 100 clusters of 50 people aged ≥50 years through probability proportionate to size sampling, using the electoral roll as the sampling frame. Households were selected within clusters using a modified compact segment sampling method [13]. The village leaders produced a sketch map of the polling area. The polling area was divided into segments each including approximately 50 people aged ≥50 years. One segment was chosen at random by drawing lots and all households in the segment were included in the sample sequentially, until 50 people aged ≥50 years were identified. If the segment did not include 50 people aged ≥50 years then an additional segment was chosen at random and sampling continued.

The enumeration team visited households, assisted by a village guide, and invited all eligible participants aged ≥50 years to the examination clinic, held at a convenient place in the cluster over the subsequent two days. Eligible participants were defined as those aged ≥50 years resident in the cluster (i.e. living there at least 6 months per year) who had slept in the house either the night before or were planning on sleeping in the house that night. If an eligible person was absent then the survey team revisited the household at least twice.

The six-year follow-up assessment was initiated by an Advance Team who visited homes of baseline participants and confirmed their identity using National Identity cards. The two advance teams comprised of one nurse, one field officer and a driver or public transport. During this visit they located individuals with assistance from the guide, phone numbers when available and previously recorded GPS locations using a Garmin Oregon 450 Satellite Navigation device. In addition, the team explained details of the examination and obtained written/thumb print informed consent as well as informing participants about location and time of examination [11].

### Data collection

Comprehensive data were collected at baseline and follow-up, using comparable methods, including slit lamp examination by an ophthalmologist at both time points. Details of data collection are available elsewhere [11, 14] with specific details provided here for the current analyses.

#### Diabetes mellitus

A single random finger-prick blood sample was taken to measure glucose (Accutrend GC system) at baseline and at follow-up. At follow-up, in addition, subjects with a random blood sugar greater than 11.1 mmol/L (International Diabetes Federation (IDF) guidance at time of baseline study), those with known DM (regardless of random measure), evidence of DR on retinal imaging and a subset (chosen randomly within each cluster) with random glucose between 7 and 11 mmol/L had an additional capillary blood HbA1C (A1C Now+, Bayer).

#### Visual acuity (VA)

Two ophthalmic nurses measured presenting VA, which was defined as the number of letters read correctly without glasses if the participant did not have glasses or with distance glasses if they had them. Each eye was tested separately at 4 m using a reduced Logarithm of the Minimal Angle of Resolution (LogMAR) tumbling ‘E’ chart [15] in a well illuminated area. If the subject’s vision was too poor to read any letters on the chart at four meters, then the subject was tested at one meter, then counting fingers, hand movements, light perception or no light perception.

#### Fundus photography

Pupil dilation was performed using one drop of tropicamide 1% and one drop of phenylephrine 2.5%. The participants had two non-stereoscopic digital 45^0^ fundus photographs taken per eye by an ophthalmic clinical officer using a TRC-NW6S Non-Mydriatic Retinal Camera with 10 megapixel Nikon D80 (TopCon®) at baseline and a Haag-Streit DRS CentreVue + at follow-up. One image was centred on the optic disc while the other was centred on the macula.

#### Anthropometric data collection

At baseline and follow-up, a nurse recorded the blood pressure of participants three times on the right arm of the participant, at least five minutes apart after an initial period of five minutes of rest using the Omron digital automatic monitor (model HEM907). Weight was measured to the nearest kilogram using standard scales (Seca 761 scales) after the participant had removed all heavy clothing and shoes. Height was measured to the nearest centimetre while the participant stood without shoes using a standardized stadiometer (Leicester Height Measure). For weight and height the average of two readings was recorded. Waist and hip circumferences were measured with a tape to the nearest centimetre.

#### Interviews

Participants were interviewed by trained nurses. Information was collected on demographic data, education and asset ownership. People were asked whether their mother tongue was “Kikuyu”, “Kalenjin” (the two largest ethnic group in Nakuru County) or “other” to assign ethnicity. Information was also collected on health behaviour (smoking, alcohol use) and health status (diagnosis of diabetes or hypertension, family history and their treatment).

#### Grading of retinal images

Retinal images were forwarded to the Retinal Grading Centre at Moorfields Eye Hospital Reading Centre (MEHRC) London for grading DR. All images supplied by the Nakuru Eye Study Group, regardless of quality, were sent for grading. No manipulation of the images was allowed while grading, other than using grey-scale for viewing the images. All images were first categorized for quality as excellent, good, borderline and ungradeable.

Next, the photographs were graded for DR based on the UK National Guidelines on Screening for Diabetic Retinopathy [16]. Each eye was classified for all people with diabetes as: no DR, mild NPDR, moderate NPDR, severe NPDR or proliferative DR, based on the following criteria:

- No DR - no changes characteristic of diabetic retinopathy visible on the images.
- Mild non-proliferative DR (NPDR) - micro aneurysms (MAs) and retinal haemorrhages only were seen.
- Moderate NPDR - in addition to MAs multiple deep, round or blot haemorrhages were noted.
- Severe NPDR - the presence of features of NPDR plus cotton wool spots. In this scenario the grader was asked to search for vascular features of DR, such as venous loop, venous beading and Intra-retinal micro-vascular abnormality (IRMA). If these were found, severe NPDR was graded.
- Proliferative DR (PDR) – as above, with new vessels on the disc (NVD) new vessels elsewhere (NVE), pre-retinal or vitreous haemorrhage or pre-retinal fibrosis ± tractional retinal detachment were seen.

All images were graded by the senior grader. In case of difficulties, the adjudicator (TP) adjudicated the images. The adjudicator also looked at a random selection of 5% of images to ensure quality control. Data were entered onto Excel and checked for consistency by a data monitor. Grading methods were the same at baseline and follow-up.

### Data analysis

#### Definitions and statistical analyses

DM was defined as per WHO standards for population-based studies: reported current medication (tablets or insulin) or; diet control for diabetes or; random blood glucose level ≥11 · 1 mmol/L [17]. At follow up the definition included HbA1C when a result was possible. HbA1C of > =7 · 0 was taken as confirmation of DM and if <7 · 0 DM was excluded. An HbA1C result superseded other measures of DM apart from self-reported and on medication, in which case an HbA1C of <7 · 0 was taken as well controlled DM and HbA1C > =7 · 0 of poorly controlled DM.

A continuous socio-economic score (SES) was produced for each participant using principal component analysis based on asset ownership, household type and education [18]. The score was divided into quartiles to categorize the study participants into four socioeconomic groups with a higher score representing higher SES. Body Mass Index (BMI) was calculated as height (meters)/weight (kilograms)^2^. The clusters were defined as *rural or urban* according to the classification used by the District Health Statistics office [19].

All participants who had complete examinations at baseline who did not have DM or did not have DR were considered “at-risk” for incident DM or DR, respectively. Follow-up status at 6 years was categorised as i) Found and examined; ii) Found and not examined; iii) Deceased; iv) Moved away; or v) Unknown.

Statistical analysis was performed using STATA v13 (Stata Corp). All analysis accounted for the cluster survey design using Taylor linearized variance estimation to calculate standard errors.

Pearson chi-squared tests, corrected for the survey-design were used to calculate *p*-values in order to assess differences between participants seen and those lost to follow-up (LTFU), and between those known to have died and with unknown outcome status. Those who were deceased were then excluded. Those followed up but without complete records for all covariates at baseline were also excluded. An inverse probability-weighting (IPW) model [20] was developed to allow estimation of cumulative incidence while accounting for those LTFU. Multivariable logistic regression was used to identify independent baseline covariates associated with LTFU. Covariates for which there was evidence of univariable association with being LTFU (*p* < 0.1) were kept in a multivariable model. From this final model, the probability of being followed-up was estimated, based on the presence or absence of each of these baseline covariates. The inverse of this probability formed the weighting to be applied in order to account for those LTFU. The final step was to remove those individuals LTFU from the cohort, so that all subsequent analysis would be performed on only those with complete outcome records, with IPW applied to account for those LTFU. A sensitivity analyses for this approach involved a complete records analysis (i.e. only including those people who had complete records for outcome and all variables in the analysis).

The six-year cumulative incidence of each outcome was calculated by dividing the number of events identified at 6-year follow-up by the number of people at risk at the beginning of follow-up. 95% confidence intervals were estimated assuming a Poisson distribution of events. This analysis was done for the population overall, and stratified by key covariates. Age-adjusted risk ratios of the outcomes (DM and DR respectively) were estimated for each covariate using a Poisson regression model with robust error variance to allow for the clustered design and including IPW. For multivariable analysis, an initial model was fitted that included those variables associated with outcome in age-adjusted analysis (Wald *p*-value <0.05). A backward stepwise approach was applied to obtain a final multivariable model, removing variables with *p* > 0.05. Estimates were weighted to allow for any bias due to loss to follow up by weighting using inverse probability weights as described above. The six-year cumulative incidence was then used to estimate the expected number of new DR cases per year by multiplying the six-year incidence by the estimated Kenyan population and dividing by six, with the assumption that cumulative incidence was constant over time. Annual cumulative incidence was also estimated separately for men and women and in ten-year age categories (50–59, 60–69, 70–79 and 80 + .

## Results

### Diabetes mellitus

**Fig. 1:**
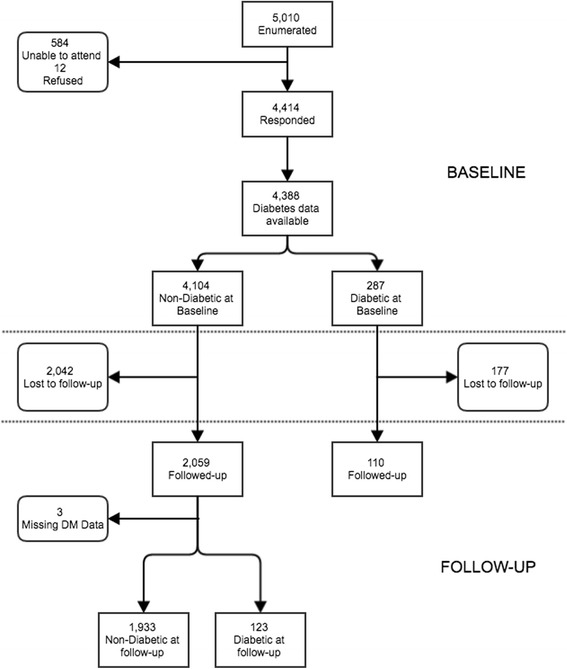
Flowcharts of participant Diabetes Mellitus status in the Nakuru Eye Disease Cohort Study

At baseline, 4414 participants aged ≥50 years underwent complete examination (response rate of 88 · 1%) and 4388 (99 · 4%) had DM status data available, of whom 287 (6 · 5%) were diagnosed with DM (Fig. 1). Of the 4,101who had no DM at baseline, 2059 (50 · 2%) were followed-up at six-years. Complete DM status data at follow-up were available on 2056 (99 · 9%) participants, of whom 123 (6 · 0%) were newly diagnosed with DM at six-year follow-up (Fig. 1).

Baseline characteristics of individuals with known baseline DM status data who were re-examined at follow-up (participants) and those who were LTFU are shown in Table 1. There was strong evidence that those who were LTFU were less likely to be Kikuyu or Kalenjin speakers and more likely to be from urban areas (*p* < 0.001). The mean follow-up time of all participants was 5 · 6 (SD 0 · 6) years and the median was 5 · 5 (interquartile range (IQR): 5 · 0–6 · 1) years (referred to as “six-year cumulative incidence” from here on).

**Table 1:** Baseline characteristics of all individuals with a known baseline DM status subdivided by their follow-up category (participant, non-participant) at 6-year follow-up (*N* = 4388)

			Participants	Non-participants or not included in analysis			
Baseline characteristics		Missing values	Followed-up *n* = 2166 (49.4%)	Not followed-up Alive/Unknown/DM status missing *n* = 1814 (41.3%)	*p*-value*	Deceased *n* = 408 (9.3%)	*p*-value**
Age in years, mean (SD)		0	62.7 (9.4)	62.6 (10.4)	0.84	71.6 (12.8)	<0.001
Systolic BP in mmHg, mean (SD)		12	139.5 (23.5)	140.8 (24.8)	0.16	147.4 (30.3)	<0.001
Diastolic BP in mmHg , mean (SD)		12	82.6 (13.0)	83.3 (13.6)	0.19	82.7 (16.5)	0.94
Random Blood Glucose, mean (SD)		92	5.1 (2.3)	5.3 (2.3)	0.12	5.7 (3.7)	<0.001
Sex, % (n)	Female	0	1025 (47.3%)	841 (46.4%)	0.59	236 (57.8%)	<0.001
	Male		1141 (52.7%)	973 (53.6%)		172 (42.2%)	
BMI, % (n)	Underweight (<18.5 kg/m^2^)	42	267 (12.4%)	250 (14.0%)	0.52	99 (25.0%)	<0.001
	Normal (18.5–24.99 kg/m^2^)		1091 (50.5%)	882 (49.2%)		199 (50.3%)	
	Overweight (25–29.99 kg/m^2^)		506 (23.4%)	419 (23.4%)		66 (16.7%)	
	Obese (30 + kg/m^2^)		295 (13.7%)	240 (13.4%)		32 (8.1%)	
Vision status impaired (<6/12 better eye), % (n)	Normal	17	1985 (91.7%)	1635 (90.8%)	0.36	306 (75.4%)	<0.001
	Impaired		180 (8.3%)	165 (9.2%)		100 (24.6%)	
Tribe, % (n)	Kikuyu	0	1393 (64.3%)	1079 (59.5%)	<0.001	283 (69.4%)	0.09
	Kalenjin		540 (24.9%)	380 (20.9%)		92 (22.5%)	
	Other		233 (10.8%)	355 (19.6%)		33 (8.1%)	
Education, % (n)	None	1	193 (8.9%)	204 (11.3%)	0.03	26 (6.4%)	<0.001
	Primary		687 (31.7%)	588 (32.4%)		179 (43.9%)	
	Secondary		1069 (49.4%)	806 (44.5%)		174 (42.6%)	
	Higher		217 (10.0%)	215 (11.9%)		29 (7.1%)	
Residence, % (n)	Rural	0	1636 (75.5%)	1014 (55.9%)	<0.001	302 (74.0%)	0.59
	Urban		530 (24.5%)	800 (44.1%)		106 (26.0%)	
SES Quartile, % (n)	Lower	22	514 (23.8%)	442 (24.5%)	0.01	136 (33.3%)	0.003
	Middle lower		591 (27.4%)	404 (22.4%)		96 (23.5%)	
	Middle upper		557 (25.8%)	438 (24.3%)		97 (23.8%)	
	Upper		495 (22.9%)	517 (28.7%)		79 (19.4%)	
Smokers, % (n)	Never	0	1503 (69.4%)	1322 (72.9%)	0.007	255 (62.5%)	0.02
	Former		163 (7.5%)	145 (8.0%)		33 (8.1%)	
	Current		500 (23.1%)	347 (19.1%)		120 (29.4%)	
Alcohol, % (n)	Never	5	882 (40.8%)	704 (38.9%)	0.10	117 (28.7%)	<0.001
	Former		942 (43.6%)	772 (42.6%)		221 (54.2%)	
	Current		339 (15.7%)	336 (18.5%)		70 (17.2%)	

*P-value for association between the baseline characteristic and the odds of having a known DM status observation at follow up, amongst all participants identified as non-diabetic at baseline and not known to be deceased at follow up

**P-value for association between the baseline characteristic and the odds of dying during the follow up period, amongst all participants identified as non-diabetic at baseline and either followed up or known to be deceased at follow up (i.e. excluding the group who were not followed up)

Of the 123 participants who developed incident DM by six-years, 64 diagnoses were self-reported and 59 were based on blood sugar readings. Of the 64 self-reported subjects with DM, 35 (54 · 7%) had random and HbA1C readings within normal limits and were considered “controlled DM”, and the remaining 29 were “uncontrolled DM” (10 had high HbA1C only, three had elevated random glucose data only (no HbA1C data) and 16 had both a high random glucose and HbA1C). Consequently, the cumulative incidence of DM was estimated at 61 · 0 per 1000 (95% CI: 50 · 3,73 · 7) in people aged ≥50 years when corrected for loss to follow-up. The incidence of DM decreased with age in both men and women, and was similar across the sexes (Table 2). Based on recent census data, the number of incident cases was estimated by extrapolating to the Kenyan population aged ≥50 years (Table 3), assuming an equal incidence per year over the study period. In the population of 1 · 6 million people in Nakuru, Kenya, approximately 150,000 individuals are aged ≥50 years, and of these approximately 1650 will develop DM each.

**Table 2:** Age-gender–specific 6-year cumulative incidence of diabetes mellitus among the Nakuru eye disease cohort study participants

	Male		Female		Overall	
Age group (years)	N (Cases / at risk)	Risk per 1000/6 years (95% CI)^a^	N	Risk per 1000/6 years (95% CI)^a^	N	Risk per 1000/6 years (95% CI)^a^
Diabetes Mellitus (*N* = 2056)						
50–59	24 / 393	63.7(43.0,93.3)	37 / 544	69.6(49.2,97.7)	61 / 937	67.1(52.2,85.8)
60–69	19 / 328	62.3(39.2,97.6)	22 / 326	67.0(42.6103.8)	41 / 654	64.7(46.3,89.5)
70–79	8 / 180	48.2(22.2101.5)	9 / 151	57.5(29.9107.8)	17 / 331	52.6(33.0,82.7)
80+	3 / 66	40.3(12.3123.9)	1 / 68	11.9(1.6,84.9)	4 / 134	25.8(9.6,67.5)
All ages	54 / 967	58.6(44.7,76.4)	69 / 1089	63.0(48.8,81.1)	123 / 2056	61.0(50.3,73.7)
Diabetic Retinopathy – among those without DM and without DR at baseline (*N* = 1421)						
50–59	5 / 297	24.6(8.5,68.9)	7 / 394	20.0(7.8,50.3)	12 / 691	22.0(11.0,43.4)
60–69	5 / 237	22.9(9.8,53.0)	1 / 229	3.9(0.5,28.5)	6 / 466	13.3(5.4,32.8)
70–79	2 / 123	15.2(3.6,61.4)	0 / 89	–	2 / 212	8.6(2.1,34.8)
80 + ^b^	0 / 29	–	0 / 23	–	0 / 52	–
All ages	12 / 686	20.5(10.9,38.2)	8 / 735	11.5(4.8,27.1)	20 / 1421	15.8(9.5,26.2)
Diabetic Retinopathy – among those with DM at baseline, but without DR at baseline (*N* = 44)						
50–59	3 / 8	400.4(83.3830.7)	3 / 14	198.9(46.8556.8)	6 / 22	278.3(111.7541.7)
60–69	4 / 10	409.8(130.2763.1)	0 / 4	–	4 / 14	268.8(78.8612.2)
70–79	1 / 4	175.5(2.7943.6)	0 / 2	–	1 / 6	126.8(6.3770.0)
80 + ^b^	0 / 1	–	0 / 1	–	0 / 2	–
All ages	8 / 23	337.7(162.1573.3)	3 / 21	118.8(29.7372.5)	11 / 44	224.7(116.9388.2)

^a^Estimated using inverse probability weights to account for loss to follow up

^b^No-one with DR at follow up among 80+ group

Risk of the outcome in the 6-year follow up, adjusted for loss to follow up using inverse probability weightings

Sample sizes are small for the DR analyses, so estimates have a wide confidence interval

**Table 3:** Extrapolated number of new adults, per year, aged 50 years and over in Kenya with diabetes mellitus and diabetic retinopathy based on incidence data (adjusted to take account of loss to follow up) and estimates of the population in Kenya by age group in 2015

	Male			Female			Overall		
Age group (years)	Extrapolated number	Lower (95% CI)	Upper (95% CI)	Extrapolated number	Lower (95% CI)	Upper (95% CI)	Extrapolated number	Lower (95% CI)	Upper (95% CI)
Diabetes Mellitus									
50–59	10,710	7230	15,690	12,760	9020	17,910	23,570	18,340	30,140
60–69	5350	3370	8390	7400	4700	11,460	12,690	9080	17,560
70–79	1950	900	4100	3010	1570	5650	4880	3060	7680
80+	490	150	1500	200	30	1430	750	280	1950
All ages	17,910	13,660	23,350	22,860	17,710	29,390	40,780	33,630	49,270
Diabetic Retinopathy – among those without DM and those without DR									
50–59	4230	1460	11,860	3850	1500	9690	8020	4020	15,840
60–69	2090	890	4820	450	60	3300	2760	1110	6770
70–79	640	150	2610	–	–	–	840	200	3410
80+	–	–	–	–	–	–	–	–	–
All ages	6520	3460	12,160	4390	1850	10,340	11,100	6670	18,370
Diabetic Retinopathy – among those without DR at baseline									
50–59	1650	340	3430	1850	430	5170	3790	1520	7390
60–69	2090	660	3880	–	–	–	2840	830	6470
70–79	370	10	1980	–	–	–	620	30	3780
80+	–	–	–	–	–	–	–	–	–
All ages	4300	2070	7310	2260	570	7100	7080	3690	12,240

All are based on 2015 estimates of population

*Diabetes Mellitus*: Population at risk are all adults over 50 who do not have DM. To estimate the size of the population at risk the 2008 DM prevalence is used. Expected number of new DM diagnoses in 50+ year old individuals per year is (population at risk x risk per 1000/6 years)/(6 × 1000)

*Diabetic Retinopathy among those with no DM and without DR at baseline*: Population at risk are all adults over 50 who do not have DR. To estimate the size of the population at risk the 2008 DR prevalence is used. Expected number of new DR diagnoses in 50+ year old individuals per year is (population at risk x risk per 1000/6 years)/(6 × 1000)

*Diabetes Retinopathy among those with DM out without DR at baseline*: Population at risk are all adults over 50 who have DM but do not have DR. To estimate the size of the population at risk the 2008 DR prevalence is used. Expected number of new DR diagnoses in those 50+ year old with DM per year is (population at risk x risk per 1000/6 years)/(6 × 1000)

Sample sizes are small for the DR analyses, so estimates have wide confidence intervals

Of the 287 participants with known DM from baseline, 54 (18.8%) were known to have died, 110 (38.3%) were re-assessed and 123 (42.8%) were LTFU. All 110 people with known DM at baseline were defined as having known DM at follow-up regardless of self-report. Of these, 70 (63 · 6%) self-reported as DM at follow-up, of whom 20 (28 · 6%) were controlled. Of the 40 with known DM at baseline who did not report as having DM at follow-up, 32 (80 · 0%) were controlled. Of these, 25 had a normal random blood sugar at baseline but self-reported as DM, four did not self-report but had a high random blood sugar and three both self-reported and had a high blood sugar.

Increased risk of incident DM was associated with the following baseline variables: higher body mass index, urban dwelling, higher socioeconomic status, hypertension, and having no previous formal education (Table 4). A lower incidence was found with increasing age, former alcohol consumption and Kalenjin ethnicity. After adjustment for confounding, increasing age and higher body mass index remained associated with incident DM (Table 4).

**Table 4:** Age-adjusted and multivariable analysis of a number of baseline co-variables and incident diabetes mellitus in the Nakuru eye disease cohort study

	Study sample, *n* = 2056				
	No at risk of diabetes mellitus	Incident diabetes mellitus	Risk per 1000/6 years (95% CI)	Age adjusted risk ratio (95% CI)	Multivariable adjusted risk ratio (95% CI)^a^
Age					
50–59	937	61	67.1 (52.2–85.8)	Baseline	Baseline
60–69	654	41	64.7 (46.3–89.5)	0.96 (0.64–1.45)	1.10 (0.74–1.63)
70–79	331	17	52.6 (33.0–82.7)	0.78 (0.47–1.30)	1.05 (0.64–1.72)
80+	134	4	25.8 (9.6–67.5)	0.38 (0.14–1.05)	0.58 (0.21–1.59)
Gender					
Male	967	54	58.6 (44.7–76.4)	Baseline	–
Female	1089	69	63.0 (48.8–81.0)	1.06 (0.74–1.51)	–
BMI (5 missing values)					
Underweight	260	5	17.5 (6.1–49.2)	Baseline	Baseline
Normal	1050	29	27.4 (18.5–40.4)	1.54 (0.50–4.79)	1.54 (0.50–4.79)
Overweight	465	54	120.4 (91.6–156.8)	6.69 (2.26–19.81)	6.69 (2.26–19.81)
Obese	276	34	123.3 (85.9–173.8)	6.83 (2.28–20.49)	6.83 (2.28–20.49)
Location					
Rural	1571	79	48.6 (39.2–60.1)	Baseline	–
Urban	485	44	87.0 (63.5–118.2)	1.75 (1.20–2.56)	–
SES Quartile (9 missing values)					
Lower	504	15	28.9 (17.9–46.5)	Baseline	–
Lower middle	576	26	46.8 (31.4–69.3)	1.59 (0.87–2.89)	–
Upper middle	520	40	75.4 (54.7–103.2)	2.54 (1.47–4.36)	–
Upper	447	41	94.3 (71.7–123.1)	3.12 (1.80–5.40)	–
Smoker					
Never	1426	88	62.6 (49.9–78.2)	Baseline	–
Former	161	4	31.4 (9.4–100.2)	0.50 (0.15–1.63)	–
Current	469	31	66.8 (47.8–92.7)	1.08 (0.73–1.62)	–
Hypertension (7 missing values)					
No	1084	44	43.3 (31.6–59.2)	Baseline	–
Yes	965	78	80.3 (64.1–100.3)	1.94 (1.33–2.82)	–
Alcohol (3 missing values)					
Never	835	60	73.0 (55.6–95.2)	Baseline	–
Former	891	42	47.5 (35.3–63.7)	0.68 (0.46–1.00)	–
Current	327	20	66.7 (41.8–104.9)	0.94 (0.55–1.61)	–
Ethnic group					
Kikuyu	1308	86	68.2 (53.5–86.6)	Baseline	–
Kalenjin	530	23	42.6 (28.3–63.6)	0.62 (0.40–0.98)	–
Other	218	14	60.5 (35.6–101.1)	0.84 (0.45–1.54)	–
Education level					
No education	173	14	88.9 (56.1–13.8)	1.77(1.01–3.12)	–
Primary	665	32	45.7 (31.8–65.4)	Baseline	–
Secondary	1009	61	61.6 (46.9–80.5)	1.25 (0.80–1.95)	–
College/Uni	209	16	80.0 (48.4–129.4)	1.58 (0.85–2.95)	–

^a^For multivariable analysis, an initial model was fitted that included those variables shown to be associated with outcome in age-adjusted analysis (using a Wald test threshold *p*-value of <0.05 to indicate association). A backward stepwise approach was then applied in order to obtain a final multivariable model, removing variables with *p* > 0.05 one-by-one

### Diabetic retinopathy

**Fig. 2:**
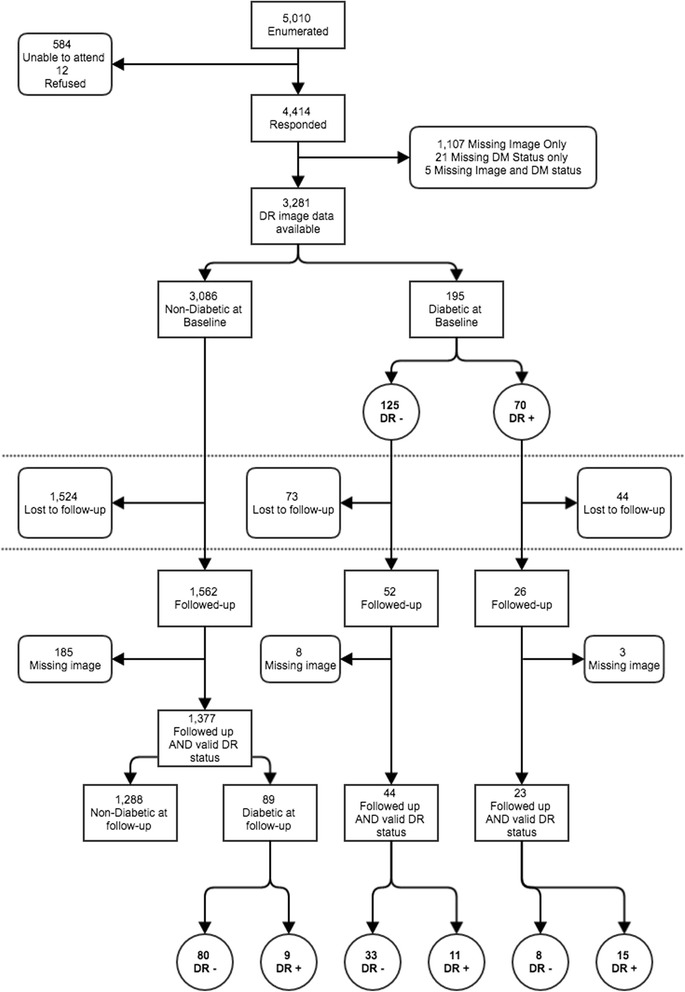
Flowcharts of participant Diabetic Retinopathy status in the Nakuru Eye Disease Cohort Study. DM: Diabetes Mellitus, DR: Diabetic Retinopathy

At baseline, 4414 participants aged ≥50 years underwent complete examination (response rate of 88 · 1%) and 3281 (74 · 3%) had DR image data available, of whom 195 (5 · 9%) were diagnosed with DM at baseline (Fig. 2). Of these 195 participants, 70 had DR at baseline. At follow-up, 78 (40 · 0%) of the 195 participants with baseline DM were seen, and 1562 (50 · 6%) of the 3086 participants without DM at baseline.

The baseline characteristics of those with complete data for analysis (participants with a known DR status at follow-up, based on retinal images), and those for whom data was incomplete were similar. However a higher proportion of those who had impaired vision at baseline were not included in the DR incidence analysis, either due to LTFU or ungradeable retinal images (Table 5).

**Table 5:** Baseline characteristics of all individuals with a known baseline DR status, subdivided by their follow-up category (participant, non-participant) at 6-year follow-up (*N* = 3281)

			Participants	Non-participants or not included in analysis			
Baseline characteristics		Missing values	Followed-up *n* = 1444 (44.0%)	Not followed-up Alive/Unknown/DM status missing *n* = 1555 (47.4%)	*p*-value*	Deceased *n* = 282 (8.6%)	*p*-value**
Age in years, mean (SD)		0	61.4 (8.6)	62.7 (10.2)	0.015	69.6 (11.8)	<0.001
Systolic BP in mmHg, mean (SD)		8	138.6 (23.6)	140.4 (24.1)	0.084	145.2 (29.6)	0.001
Diastolic BP in mmHg , mean (SD)		8	82.9 (13.0)	82.9 (13.1)	0.865	82.6 (16.4)	0.72
Random Blood Glucose, mean (SD)		69	5.1 (2.3)	5.2 (2.3)	0.10	5.5 (3.0)	0.04
Sex, % (n)	Female	0	701 (48.5%)	746 (48.0%)	0.78	172 (61.0%)	<0.001
	Male		743 (51.5%)	809 (52.0%)		110 (39.0%)	
BMI, % (n)	Underweight (<18.5 kg/m^2^)	11	166 (11.5%)	220 (14.2%)	0.07	68 (24.2%)	<0.001
	Normal (18.5-24.99 kg/m^2^)		738 (51.2%)	757 (48.9%)		138 (49.1%)	
	Overweight (25-29.99 kg/m^2^)		343 (23.8%)	360 (23.3%)		51 (18.1%)	
	Obese (30 + kg/m^2^)		194 (13.5%)	211 (13.6%)		24 (8.5%)	
Vision status impaired (<6/12 better eye), % (n)	Normal	4	1375 (95.3%)	1415 (91.1%)	<0.001	233 (82.9%)	<0.001
	Impaired		68 (4.7%)	138 (8.9%)		48 (17.1%)	
Tribe, % (n)	Kikuyu	0	912 (63.2%)	891 (57.3%)	0.001	191 (67.7%)	0.28
	Kalenjin		358 (24.8%)	354 (22.8%)		63 (22.3%)	
	Other		174 (12.0%)	310 (19.9%)		28 (9.9%)	
Education, % (n)	None	1	129 (8.9%)	168 (10.8%)	0.004	20 (7.1%)	0.001
	Primary		425 (29.4%)	518 (33.3%)		116 (41.1%)	
	Secondary		739 (51.2%)	685 (44.1%)		121 (42.9%)	
	Higher		151 (10.5%)	183 (11.8%)		25 (8.9%)	
Residence, % (n)	Rural	0	1062 (73.5%)	863 (55.5%)	<0.001	199 (70.6%)	0.43
	Urban		382 (26.5%)	692 (44.5%)		83 (29.4%)	
SES Quartile, % (n)	Lower	16	310 (21.6%)	385 (24.9%)	0.003	87 (30.9%)	0.03
	Middle lower		399 (27.7%)	354 (22.9%)		63 (22.3%)	
	Middle upper		386 (26.8%)	380 (24.6%)		72 (25.5%)	
	Upper		343 (23.9%)	426 (27.6%)		60 (21.3%)	
Smokers, % (n)	Never	0	981 (67.9%)	1115 (71.7%)	0.02	167 (59.2%)	0.02
	Former		113 (7.8%)	139 (8.9%)		23 (8.2%)	
	Current		350 (24.2%)	301 (19.4%)		92 (32.6%)	
Alcohol, % (n)	Never	3	586 (40.6%)	584 (37.6%)	0.06	82 (29.1%)	0.001
	Former		624 (43.2%)	664 (42.8%)		147 (52.1%)	
	Current		233 (16.1%)	305 (19.6%)		53 (18.8%)	

*P-value for association between the baseline characteristic and the odds of having a valid DM observation at follow up, amongst all participants identified as having no diabetes at baseline and not known to be deceased at follow up

**P-value for association between the baseline characteristic and the odds of dying during the follow up period, amongst all participants identified as no-DM at baseline and either followed up or known to be deceased at follow up (i.e. excluding the group who were not followed up)

Of the 1562 people without either DM or DR at baseline, 1377 (88 · 1%) had complete follow-up DR status data. Of these, 89/1377 (6 · 5%) were newly diagnosed with DM; and 9 (10 · 1%) had incident DR. A further 11 incident cases of DR were seen in the 44 participants with DM but no DR at baseline (Fig. 2). Therefore, in total, 20 participants developed DR during follow up, giving a corrected cumulative incidence of 15 · 8 per 1000 (95% CI: 9 · 5–26 · 3), Table 2. This equates 15,800 cases in the population aged ≥50 years per year per million. In the population of 1 · 6 million people in Nakuru, Kenya, approximately 150,000 individuals are aged ≥50 years, and of these approximately 1650 will develop DM each year and 450 will develop DR each year.

Among subjects with known DM at baseline, the corrected cumulative incidence of DR is 224 · 7 per 1000 (95% CI: 116 · 9–388 · 2) (Table 2). Similarly to DM, the incidence of DR decreased with increasing age (Table 2). Of the 20 incident cases of DR, seven had sight-threatening DR (STDR) of whom two cases were proliferative DR (five with severe retinopathy), four cases had moderate DR and nine mild DR (Table 2).

In total 23 participants with known DR at baseline were followed up and had a gradable retinal image. Of these, 15 still had signs of DR, while eight no longer had evidence of DR. Of nine with background DR at baseline, one progressed to pre-proliferative DR and the remainder either remained BDR (*n* = 2) or had no signs of DR at follow-up (*n* = 6). Of seven participants with moderate non-proliferative DR (NPDR) at baseline, three progressed to proliferative DR (PDR). One participant with severe NPDR at baseline developed PDR and one remained Severe NPDR. Of five with PDR at baseline, one regressed to Moderate NPDR (having undergone pan-retinal photocoagulation) and the other four remained PDR, of whom two had evidence of laser treatment.

Multivariable analysis of incident DR (Table 6) was not conducted due to the small number of participants in the category (*n* = 20). However, the age-adjusted risk ratio suggested a correlation between increasing incidence of DR and higher BMI, urban dwelling and higher socioeconomic status. No conclusions can be drawn from this due to the wide 95% confidence intervals.

**Table 6:** Age-adjusted analysis the association between a number of baseline co-variables and incident DR amongst those DR free at baseline in the Nakuru eye disease cohort study

	Study sample, *n* = 1421			
	No at risk of diabetic retinopathy	Incident diabetic retinopathy	Risk per 1000/6 years (95% CI)	Age adjusted risk ratio (95% CI)
Age				
50–59	691	12	22.0 (11.0–43.4)	Baseline
60–69	466	6	13.3 (5.4–32.8)	0.6 (0.2–2.0)
70–79	212	2	8.6 (2.1–34.8)	0.4 (0.1–1.9)
80+	52	0	–	–
Gender				
Male	686	12	20.5 (10.9–38.2)	Baseline
Female	735	8	11.5 (4.8–27.1)	0.5 (0.2–1.5)
BMI (2 missing values)				
Underweight	166	1	5.3 (0.7–36.6)	Baseline
Normal	727	5	8.1 (3.3–19.5)	1.5 (0.2–13.0)
Overweight	336	11	39.6 (20.3–75.9)	6.4 (0.8–53.6)
Obese	190	3	12.9 (4.1–40.2)	2.0(0.2–21.9)
Location				
Rural	1053	12	11.6 (6.6–20.4)	Baseline
Urban	368	8	23.5 (10.3–52.7)	1.8 (0.7–4.4)
SES Quartile (6 missing values)				
Lower	309	1	3.0 (0.4–21.8)	Baseline
Lower middle	394	2	4.9 (1.2–19.4)	1.6 (0.4–6.9)
Upper middle	380	9	25.1(13.1–47.5)	7.5 (1.0–58.1)
Upper	332	8	29.3 (13.3–63.3)	8.3 (1.0–66.9)
Smoker				
Never	964	16	18.3 (10.0–33.1)	Baseline
Former	113	0	–	–
Current	344	4	14.3 (5.4–37.5)	0.8 (0.3–2.6)
Hypertension (2 missing values)				
No	764	8	15.0 (6.0–36.7)	Baseline
Yes	655	12	16.9 (9.3–30.3)	1.2 (0.4–3.6)
Alcohol (1 missing value)				
Never	580	8	15.3 (6.5–35.6)	Baseline
Former	611	9	15.0 (7.4–30.1)	1.1 (0.4–3.3)
Current	229	3	19.0 (4.1–83.5)	1.3 (0.2–7.9)
Ethnic group				
Kikuyu	895	14	16.9 (10.3–27.8)	Baseline
Kalenjin	357	3	8.5 (1.9–36.9)	0.5 (0.1–2.3)
Other	169	3	22.6 (7.2–69.1)	1.1 (0.4–3.0)
Education level				
No education	123	4	42.8 (15.3–114.2)	Baseline
Primary	422	0	–	–
Secondary	726	14	21.7(11.9–39.0)	0.6(0.2–1.9)
College/Uni	150	2	12.6(3.0–51.6)	0.3(0.0–1.8)

For multivariable analysis, an initial model was fitted that included those variables shown to be associated with outcome in age-adjusted analysis (using a Wald test threshold *p*-value of <0.05 to indicate association). A backward stepwise approach was then applied in order to obtain a final multivariable model, removing variables with *p* > 0.05 one-by-one

## Discussion

This population-based cohort study of people aged 50+ in rural Kenya is the first, to our knowledge, to assess the incidence of DM and DR in SSA. The six-year cumulative incidence of DM in this study was 61 cases per 1000, equating to approximately 10 new cases per 1000 of population aged ≥50 per year. Longo-Mbenza et al. investigated the incidence of type 2 DM in a prospective cohort of 807 subjects of Central Africans aged ≥40 years over a four-year period, all of whom had no DM at basline [2]. During the follow up, there were 93 incident DM cases (11 · 5%), corresponding to an incidence of 29 (95% CI 15-43) per 1000 persons per year, considerably higher than our estimated cumulative incidence. Motola et al. investigated the incidence of DM in a prospective cohort of 563 South African Indians with no-DM aged 15 years or greater over a ten-year period. During the follow up period there were 91 (16.2%) incident cases of DM, corresponding to an incidence (age and sex-adjusted) cumulative incidence of 8.3 per 1000 persons per year [21]. This latter estimate was more in line with ours, but included a much younger population.

The six-year cumulative incidence of DR among persons, 50 years and over, with known DM was 225 cases per 1000 (95%CI: 116 · 9388 · 2). There is minimal comparable data available for SSA. One systematic review of 62 studies that reported the prevalence or incidence of DR in SSA [6] found few high-quality population-based studies and the majority were hospital or clinic based surveys. Two cohort studies of DR have been conducted in SSA. Sixty-four patients with insulin-dependent (Type 1) DM (IDDM) in Soweto, South Africa were followed over a 10-year period between 1982 and 1992. In those subjects seen at 10 years, prevalence of DR had increased from 6 to 52% and PDR from 0 to 3%, but no incidence data was reported [22]. In a two-year prospective cohort study of DR in Malawi, 357 subjects were systematically sampled from two primary care diabetes clinics, and 295 participants were followed up. The incidence of any DR over the follow-up period was 380 per 1000 of population over two years [23]. Two of the leading cohort studies of eye disease from high-income settings, the Blue Mountains Study in Australia and The Wisconsin Epidemiologic Study of Diabetic Retinopathy (WESDR) in the United States in which comparable methodology was used show estimates of incident DR from Nakuru, Kenya are more than twice that of the findings from Wisconsin and three times that of the Blue Mountains Study. The Blue Mountains Study reported 222 cases per 1000 over five years (44 per 1000/year) and the WESDR study reported 327 cases per 1000 over a four-year period (82 per 1000/year).

In a prospective cohort in southern Malawi, sampled from two primary care diabetes clinics, the 2-year incidence of sight-threatening DR (STDR) amongst 357 subjects for subjects with no DR, BDR, and PDR at baseline was 2 · 7% (95% confidence interval [CI], 0 · 1–5 · 3), 27 · 3% (95% CI, 16 · 4–38 · 2), and 25 · 0% (95% CI, 0–67 · 4), respectively [23].

The sample of participants with DR at both study time points in the Nakuru cohort was too small to draw conclusions on the progression of DR. However, 23 participants over the six year follow up period had a DR assessment at baseline and follow-up, of whom four progressed from non-STDR to STDR and of five with STDR at baseline, one recovered to non-STDR and the other four remained with STDR [23].

### Strengths

This study is one of the first reports of DM and DR from a population-based sample in SSA. The sampling methodology ensures that the data is representative for the population over 50 and minimised bias by sampling from the community rather than from hospitals or clinics. Retinal image data were collected for DR analysis and images were independently graded at Moorfields Eye Hospital Reading Centre.

### Limitations

The definition of DM used in this study was based on a single, non-fasting, capillary blood sample and did not include fasting blood glucose samples and HbA1C measures were only available at follow-up. There was a high loss to follow-up which creates potential for selection bias however statistical methods were used to adjust for this. This high LTFU was largely due to post-election violence in the region between the two study time points which led to mass displacement. The population under observation were ≥50 years and therefore the study does not estimate the incidence of DM or DR in the population under age 50 years.

### Implications

As the diabetes epidemic continues, a greater understanding is required of the resources needed and level of deployment of those resources within the health system to respond appropriately. This includes primary prevention of DM in the community, high treatment coverage of persons with DM in primary care, and inclusion of eye screening for people with DM as standard practice. With good prevalence data on DM available in many countries and regions in SSA and a growing understanding of the natural history of the diseases in different populations it should now be possible (within a wide range of confidence and based on several assumptions) to estimate the conversion in the over 50s of those without DM developing DM and DR.

Overall, DM is increasing in Africa probably related to environmental factors such as increased access to processed foods and more sedentary lifestyles; this is likely to increase in the next decade. The awareness of DM in the community is still low [3] and public campaigns to raise awareness as well as provide locally available DM screening and counselling facilities is needed. It is also important to consider detection of VTDR in the community and resourcing eye care providers with the knowledge and tools to manage patients with STDR is essential to ensure a high quality service and avoid sight loss from DM. Specific planning data for the region under investigation is provided in Table 7.

**Table 7:** Population summary for programme planning based on prevalence and incidence data from a Kenyan cohort over 50 years of age

Population at risk		
Place	Nakuru County	Kenya (National)
Total Population	1.6 Million	46 Million
Population 50 years and over	0.15 Million	4.3 Million
Diabetes Mellitus (DM)		
Prevalence (%) of DM	6.5	
Number of people over 50 with DM (needing examination of the retina every 1–2 years)	26,100	279,500
Awareness of DM within the population over 50 (%)	85	
Number of people over 50 with known DM	22,185	237,575
Number over 50 who develop new DM per 1000 of population per year	11/1000	
Number over 50 who develop new DM per sample population per year	1650	47,300
Diabetic Retinopathy (DR)		
Proportion (%) of people over 50 with DM who have DR	35.9	
Number of people over 50 with DR	9400	100,340
Number over 50 who develop new DR per 1000 of population per year	3/1000	
Number over 50 who develop new DR of the sample population per year	450	12,900
Vision Threatening Diabetic Retinopathy (VTDR)		
Proportion of people over 50 with DM who have VTDR	13.4	
Number of people over 50 with VTDR (needing treatment)	1260	13,450
Number over 50 who develop new VTDR per 1000 of population per year	1.6/1000	
Number over 50 who develop new VTDR of the sample population per year	240	6880

Further research needs to be done to assess gaps in the patient care pathway which include:

• awareness of DM in the population;

• access / availability to relevant diagnostic and treatment services ;

• quality of diagnostic and treatment services;

• availability of screening for DR within diabetes and eye care;

• protocols and referral thresholds for people with or without DR;

• barriers to receiving treatment for STDR in those with known STDR

## Conclusions

In a population of 1 · 6 Million in Nakuru County, Kenya: 150,000 are 50 years and over, we estimated that 1650 people over the age of 50 develop DM per year, and 450 develop DR. The management of DM and DR is complex and requires different approaches at different levels of the healthcare system with considerable variation depending on location. For effective planning at any level, high quality information is required to effectively plan the services. This cohort provides some data to support planning and is indicative of areas that need further research.

## Abbreviations

BDR: Background diabetic retinopathy
DM: Diabetes mellitus
DR: Diabetic retinopathy
LTFU: Lost to follow up
NPDR: Non-proliferative diabetic retinopathy
PDR: Proliferative diabetic retinopathy
SSA: Sub Saharan Africa
VI: Visual impairment
VTDR: Vision threatening diabetic retinopathy
